# Curvature induction and sensing of the F-BAR protein Pacsin1 on lipid membranes via molecular dynamics simulations

**DOI:** 10.1038/s41598-019-51202-z

**Published:** 2019-10-10

**Authors:** Md. Iqbal Mahmood, Hiroshi Noguchi, Kei-ichi Okazaki

**Affiliations:** 1 Department of Theoretical and Computational Molecular Science, Institute for Molecular Science, National Institutes of Natural Sciences, Okazaki, 444-8585 Japan; 2 Institute for Solid State Physics, University of Tokyo, Kashiwa, Chiba 277-8581 Japan

**Keywords:** Computational biophysics, Membrane structure and assembly, Biological physics

## Abstract

F-Bin/Amphiphysin/Rvs (F-BAR) domain proteins play essential roles in biological processes that involve membrane remodelling, such as endocytosis and exocytosis. It has been shown that such proteins transform the lipid membrane into tubes. Notably, Pacsin1 from the Pacsin/Syndapin subfamily has the ability to transform the membrane into various morphologies: striated tubes, featureless wide and thin tubes, and pearling vesicles. The molecular mechanism of this interesting ability remains elusive. In this study, we performed all-atom (AA) and coarse-grained (CG) molecular dynamics simulations to investigate the curvature induction and sensing mechanisms of Pacsin1 on a membrane. From AA simulations, we show that Pacsin1 has internal structural flexibility. In CG simulations with parameters tuned from the AA simulations, spontaneous assembly of two Pacsin1 dimers through lateral interaction is observed. Based on the complex structure, we show that the regularly assembled Pacsin1 dimers bend a tensionless membrane. We also show that a single Pacsin1 dimer senses the membrane curvature, binding to a buckled membrane with a preferred curvature. These results provide molecular insights into polymorphic membrane remodelling.

## Introduction

F-Bin/Amphiphysin/Rvs (F-BAR) domain proteins are involved in important biological processes, such as endocytosis, exocytosis and cell motility^1^. Pacsin proteins are members of the Pacsin/Syndapin subfamily, one of six subfamilies of F-BAR proteins, and are involved in clathrin-mediated endocytosis, actin polymerization and neuronal development^1–6^. It has been shown that Pacsin proteins, as well as other F-BAR proteins, have the ability to transform the liposome membrane into tubes with different diameters^2,7–9^. In particular, Pacsin1 was shown to generate various membrane morphologies: striated tubes, featureless wide and thin tubes, and pearling vesicles^2^. The molecular mechanism of this interesting ability, however, is not well understood. Cryo-electron microscopy (cryo-EM) previously revealed that another F-BAR protein, CIP4, assembles into helical coats on the membrane upon tubule formation^7^. It also showed that the F-BAR protein tilts the long-axis of the banana-shaped structure against the cylindrical axis of the tubule to adjust itself on the narrower tubules with higher curvature^7^. Thus, assembly and curvature sensing are key processes in understanding the membrane remodelling ability.

Protein-membrane systems ranging from membrane remodelling to receptor, channel and transporter systems have been studied by molecular dynamics simulations^10–17^. All-atom (AA) simulations can provide accurate and detailed atomistic mechanisms, although they usually suffer from limitations in both system size and simulation time. The system size of F-BAR proteins and membranes with solvent molecules can easily exceed one million atoms in total (~900,000 atoms for a single Pacsin1 dimer on the membrane in our setup). The simulation time is also limited to sub-microseconds ~ a few microseconds by the system size. Therefore, it is difficult to reach a physiologically relevant scale with AA simulations. To overcome these limitations, coarse-grained (CG) models have been developed for lipids, proteins and water molecules^18–24^. With their improved energy functions, CG simulations have great promise for describing larger-scale BAR domains and membrane interactions.

Multiscale simulations that use both AA and CG simulations in an integrative manner have been successfully employed for BAR protein–membrane systems. In a series of studies, Schulten and coworkers simulated membrane bending and tubule formation by Amphiphysin N-BAR protein^25,26^ and CIP4 F-BAR protein^27^ using AA and CG simulations. Taking advantage of cryo-EM images of the lattice assembly formed by the BAR proteins, they showed that the lattice structure indeed transforms the membrane into tubes. Voth and coworkers have developed a CG model for a protein-membrane system based on a multiscale coarse-graining method that uses information from AA simulations to optimize the CG force field for a chosen CG representation^20,28^. Using the CG model, they simulated N-BAR assembly on the membrane and found that N-BAR proteins aggregate linearly on the flat membrane as well as on vesicles and tubes^29–31^. Contrary to the characteristics of the lattice assembly, the linear aggregations they observed were sparsely formed. Thus, it is still under debate how BAR proteins assemble to remodel the membrane.

**Figure 1:**
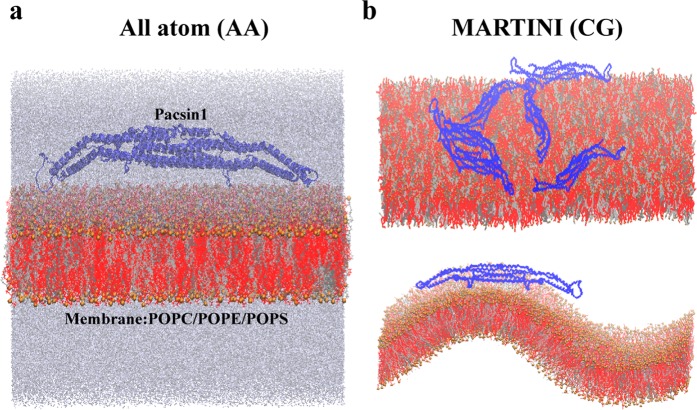
Simulation systems. Atomistic and coarse-grained MARTINI molecular dynamics systems of Pacsin1 proteins on a membrane are shown in (**a**,**b**), respectively. Pacsin1 proteins are shown in blue. The lipid bilayer membrane has three kinds of lipids (silver, 20% PC; grey, 20% PE; and red, 60% PS). Phosphate atoms are coloured in gold for clarity. Water molecules and ions are not shown in the MARTINI systems. In (**b**), flat and buckled membranes are shown in the top and bottom panels, respectively.

In this study, we investigate how Pacsin1 induces and senses curvature on the membrane by a multiscale simulation approach. First, we performed AA simulations to examine the conformational flexibility of Pacsin1 on the membrane (Fig. 1a). On the basis of the AA simulations, the elastic network model used in CG MARTINI simulations is tuned to reproduce reasonable conformations of Pacsin1. Since the CG MARTINI model has been shown to be useful for simulating protein assemblies^32–35^, we simulate Pacsin1 assembly on the flat membrane (Fig. 1b top). Based on the complex formation observed in the assembly simulation, we model the regularly assembled Pacsin1 dimers and simulate membrane bending by these dimers. We also simulate a single Pacsin1 dimer on a buckled membrane to reveal its curvature-sensing property (Fig. 1b bottom).

## Results

### Structural flexibility of Pacsin1

**Figure 2:**
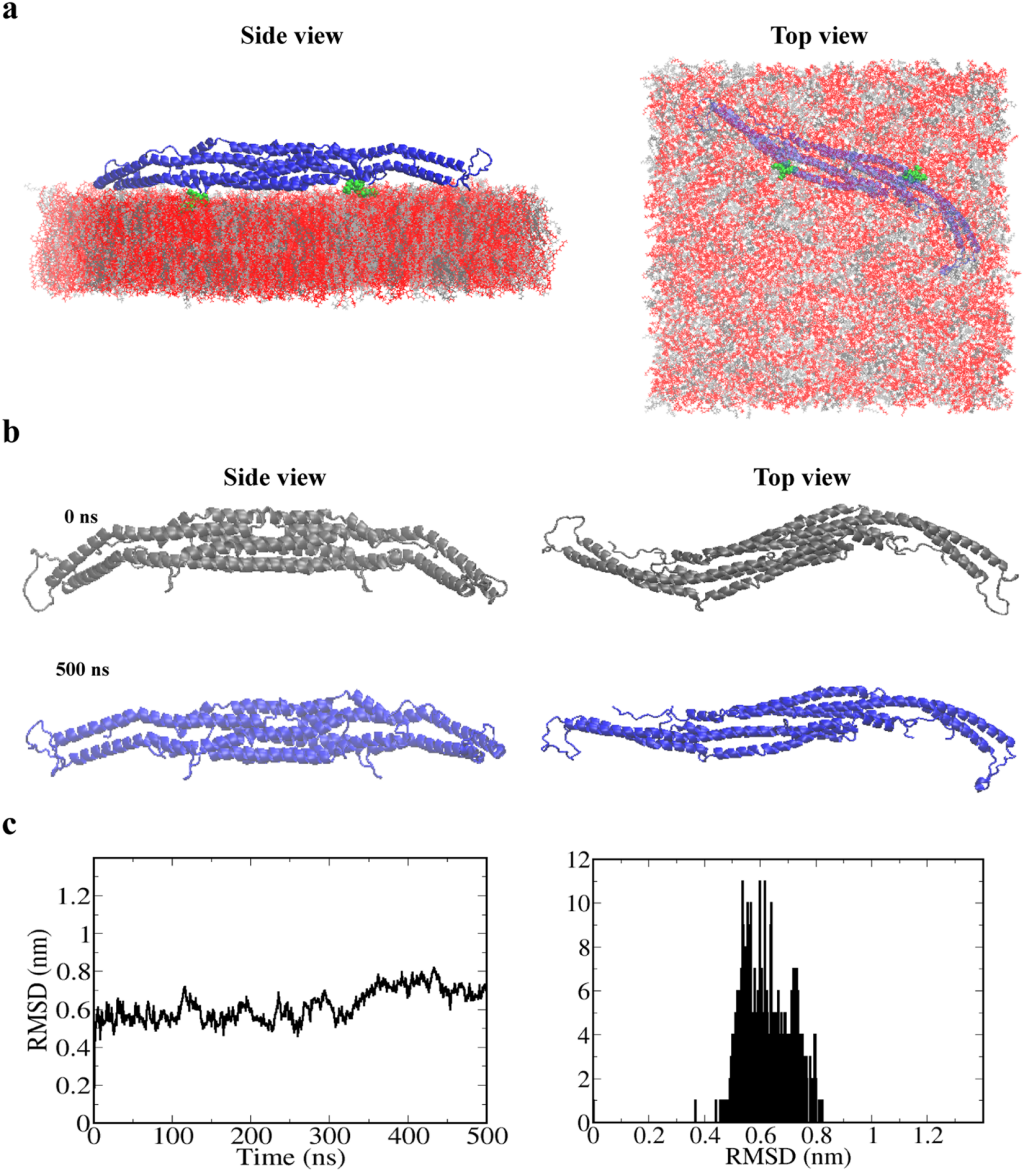
The AA simulations. (**a**) Side and top views of Pacsin1 with the wedge loops inserted into the membrane. The wedge loops are shown in green. PC, PE and PS lipids are shown in silver, grey and red, respectively. (**b**) Snapshots of the Pacsin1 structure at the start (0 ns, coloured grey) and end (500 ns, coloured blue) of the MD simulation are shown. (**c**) Time series and histogram of the RMSD of the Pacsin1 dimer structure are shown.

Pacsin1, as well as other BAR proteins, forms a banana-shaped homodimer structure (Fig. 1a). We only consider the F-BAR domain of Pacsin1 in this study, without the central linker and SH3 domain. The removal of these regions in experiments increased the membrane transformation activity^2^. In the rest of the paper, Pacsin1 means the F-BAR domain of Pacsin1. To clarify the internal structural dynamics of the Pacsin1 dimer, we performed ~500 ns long AA simulations of a single Pacsin1 dimer. In the simulation, the Pacsin1 dimer was initially placed above the mixed lipid membrane composed of phosphatidylcholine (PC), phosphatidylethanolamine (PE) and phosphatidylserine (PS). The spontaneous attachment of Pacsin1 to the membrane was observed during the first ~100 ns due to electrostatic interactions between the positive charges on the Pacsin1 concave surface and the negative charges on lipid head groups. After Pacsin1 attached to the membrane, the wedge loops were inserted into the membrane (Fig. 2a). The wedge loop is a signature of Pacsin proteins and possibly affects their assembly^8^. Negatively charged PS lipids were abundant and likely involved in Pacsin1 attachment. We observed a relative straightening of the Pacsin1 dimer structure in both the side and top views. The structure became less curved during the ~500 ns long equilibration compared to the initial crystal structure (Fig. 2b). The radius of curvature of the Pacsin1 dimer structure in the side view was initially ~25 nm and became ~50 nm and larger during the simulation (Fig. S1). Using the trajectory after Pacsin1 was fully attached to the membrane at ~100 ns (Fig. S2a), the free energy profile along the radius of curvature of the Pacsin1 structure under the influence of the membrane was calculated (Fig. S2b,c). The free energy profile shows a minimum at ~55 nm, and the free energy to bend the stable conformation back to the curved crystal structure on the flat membrane was estimated to be ~3 k_B_T, which is a rough estimate because the population around the crystal structure was not sufficient. This result is consistent with the structural variation of Pacsin2 in the side view^36^ and suggests that Pacsin proteins have structural flexibility. The highly curved structure of Pacsin1 observed in the crystal structure might result from the highly packed environment and the absence of the membrane^2^.

**Figure 3:**
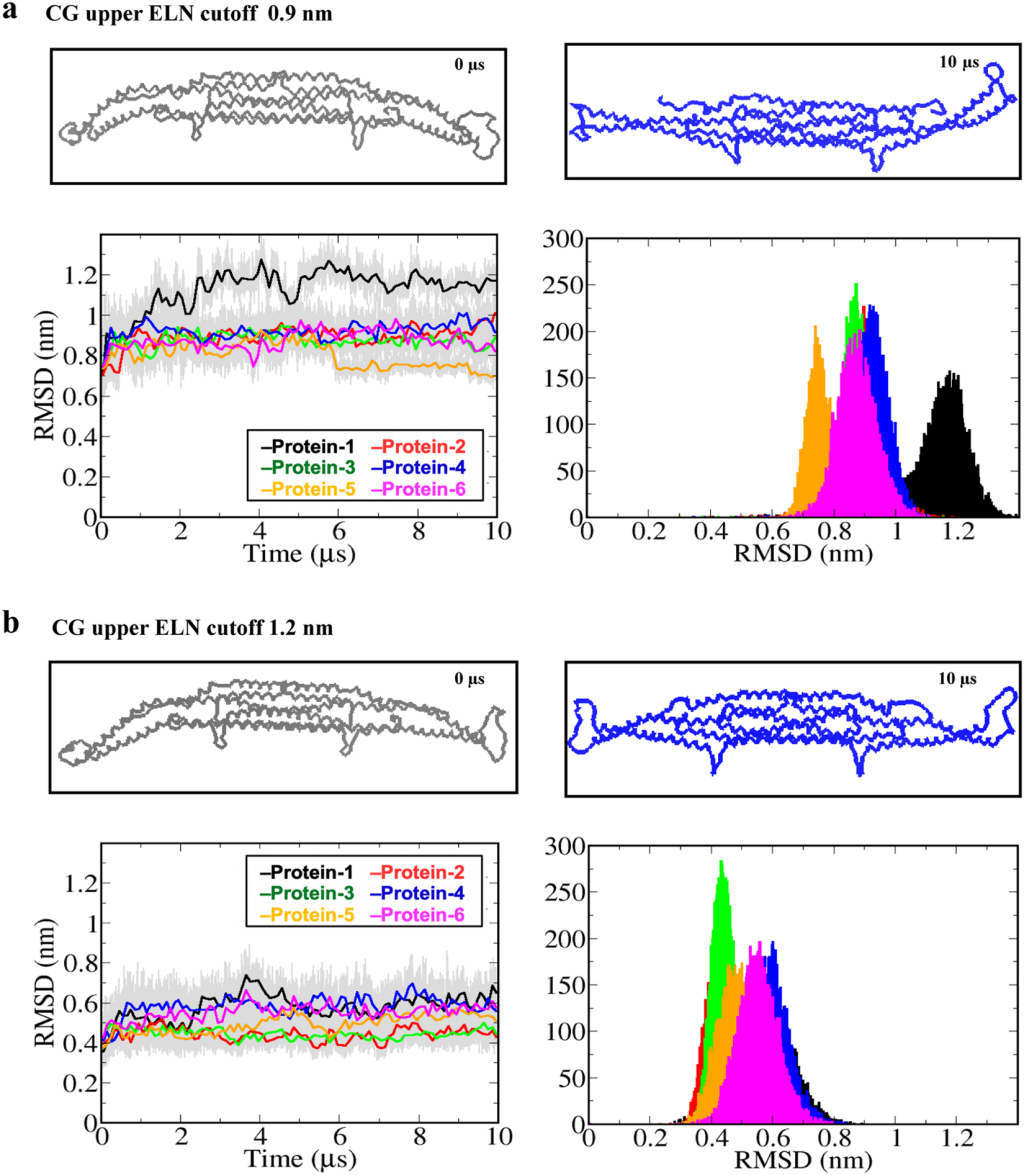
The CG MARTINI simulations. Simulation results with an elastic network upper cutoff of 0.9 nm and 1.2 nm are shown in (**a**,**b**), respectively. (Top panels) Snapshots of the Pacsin1 structure at the start (0 ns, after the initial equilibration, coloured grey) and end (10 µs, coloured blue) of the MD simulations highlight the structural changes in the CG MARTINI simulations. (Bottom panels) RMSD time series and corresponding histogram are shown with each colour representing each Pacsin1 dimer.

The above AA simulation suggested that the crystal structure solved without the membrane does not correspond to a typical conformation of Pacsin1 during its function. Thus, the AA simulation results should be input in some way into the CG model. In the CG MARTINI simulations, it is common practice to apply the elastic network potential to maintain the secondary and tertiary structures of proteins^37^. The elastic network potential and the Pacsin1-membrane interaction mainly contribute to structural ensemble of Pacsin1. We employed the crystal structure as the reference structure of the elastic network and tuned an elastic network parameter, the upper cutoff distance, by using the AA simulation results as follows. With the default elastic network parameters, the curved structure of the Pacsin1 dimer was completely lost (Fig. 3a). It seemed that helix kinks due to proline residues at Pro145 and Pro221, known as the helix breaker, were not properly maintained by the default elastic network. We tuned the elastic network by matching the structural deviation from the initial crystal structure in the CG simulation to that in the AA simulation. The root-mean-square-deviations (RMSDs) were compared between the AA simulation (Fig. 2c) and the CG simulations with different elastic network parameters (Fig. 3). In the RMSD calculations, the reference structure is the crystal structure and target atoms are all backbone atoms. Consistent with the visual inspection, Pacsin1 in the CG simulation with the default elastic network (upper cutoff distance, 0.9 nm) had higher RMSDs than those in the AA simulation. After exploring the parameter of the elastic network model, we determined that the upper cutoff distance of 1.2 nm is a suitable value on the basis of the RMSD distribution compared to the AA result (Figs 2c, 3b and Table 1). As a check of the elastic network model with the optimized cutoff, we calculated the free energy profile along the radius of curvature of the Pacsin1 structure under the influence of the membrane (Fig. S2b,c). The free energy profile agrees well with the profile from the AA simulations. We also calculated the root-mean-square fluctuations (RMSFs) of Pacsin1 from the AA and CG simulations (Fig. S3). The overall CG profile agrees well with the AA profile except for the tip-loop region, which shows a large fluctuation. The underestimation of the RMSF in the tip-loop region is due to a limitation of the elastic network model: it cannot describe large unfolding motions^38^. Although the spring constant is another parameter that can be tuned, our simulation results for different values of the spring constants show that the default value of 500 kJ mol^−1^ nm^−2^ is reasonable (Fig. S2b,c). Thus, we used the upper cutoff value of 1.2 nm and the spring constant of 500 kJ mol^−1^ nm^−2^ in the rest of the simulations in this study.

**Table 1:** Summary of MD simulations.

Simulations	Protein	Elastic Network upper cutoff, spring constant	Time	Trajectories
AA	1 Pacsin1 dimers	N/A	500 ns	2
CG flat membrane	6 Pacsin1 dimers	0.9 nm, 500 kJ mol^−1^ nm^−2^	10 µs	1
CG flat membrane	6 Pacsin1 dimers	1.1 nm, 500 kJ mol^−1^ nm^−2^	10 µs	1
CG flat membrane	6 Pacsin1 dimers	1.2 nm, 500 kJ mol^−1^ nm^−2^	5/10 µs	3
CG flat membrane	6 Pacsin1 dimers	1.2 nm, 200 kJ mol^−1^ nm^−2^	10 µs	1
CG flat membrane	6 Pacsin1 dimers	1.2 nm, 800 kJ mol^−1^ nm^−2^	10 µs	1
CG bending	14 Pacsin1 dimers	1.2 nm, 500 kJ mol^−1^ nm^−2^	2 µs	1
CG buckled	1 Pacsin1 dimers	1.2 nm, 500 kJ mol^−1^ nm^−2^	10 µs	1

### Spontaneous assembly of Pacsin1 on flat membrane

**Figure 4:**
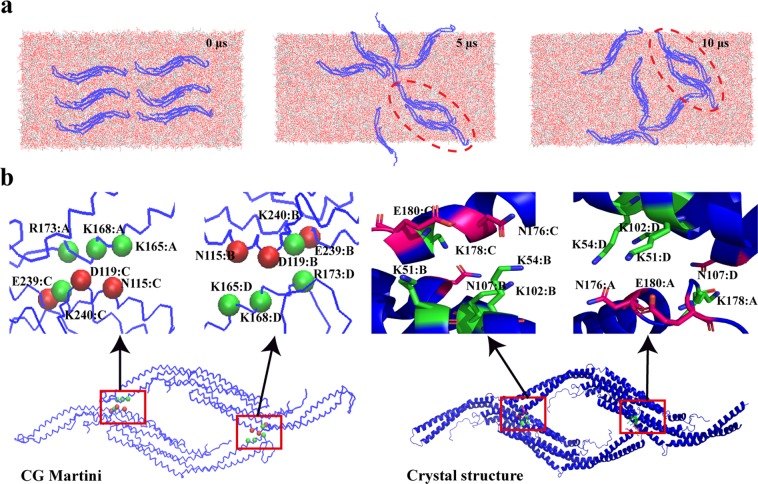
Assembly modes of Pacsin1 on a flat membrane. (**a**) Assembly process of six Pacsin1 dimers in the CG simulation is shown. (**b**) The lateral interaction formed in the simulation is compared with the interaction in the crystal structure (PDB ID: 3HAI). Two dimers interact with each other mainly through their charged residues in the MD simulation and crystal structure. The acidic and basic residues are shown in red and green, respectively. The amino acids, residue number and chain number are shown for each residue involved in the interaction.

Pacsin1 transforms the membrane into tubes or vesicles in a collective way by assembling on it. Despite its importance in membrane remodelling, little is known about how Pacsin1 assembles on the membrane. Here, we performed CG MARTINI simulations of six Pacsin1 dimers on a flat tensionless membrane. During the 10 μs long simulation, we observed two modes of Pacsin1-Pacsin1 interaction: tip-to-tip and lateral interactions (Fig. 4a). The tip-to-tip interaction involves multiple Pacsin1 dimers that interact through their tips. Number of Pacsin1 dimers involved in the interaction was up to five in our simulations, but can be more until it is restricted by the excluded volume interactions. This type of interaction was observed frequently and was relatively flexible. A similar tip-to-tip interaction was previously observed in the crystal structures of Pacsin2^2,39^. The lateral interaction was observed in two of the six Pacsin1 dimers. Once this type of interaction was formed, it was stably maintained throughout the simulation. In all three independent runs, spontaneous formation of the lateral interaction was observed (Figs 4 and S4). The lateral interaction is similar to what was observed in the crystal structure of Pacsin1 (PDB ID: 3HAI), although the specific interacting residues are not identical (Fig. 4b). In both cases, ionic interactions seem to play a major role. The two dimers did not align straight in the lateral direction but shifted to form a stable lateral interaction. A similar shifted lateral interaction was observed in the cryo-EM structure of CIP4 F-BAR proteins assembled on a tubule^7^, while a straight lateral interaction was observed in the crystal structure of Pacsin2^39^. We expect that Pacsin1 dimers can form a helical assembly via the lateral interaction^40^.

### Membrane bending induced by Pacsin1 assembly

**Figure 5:**
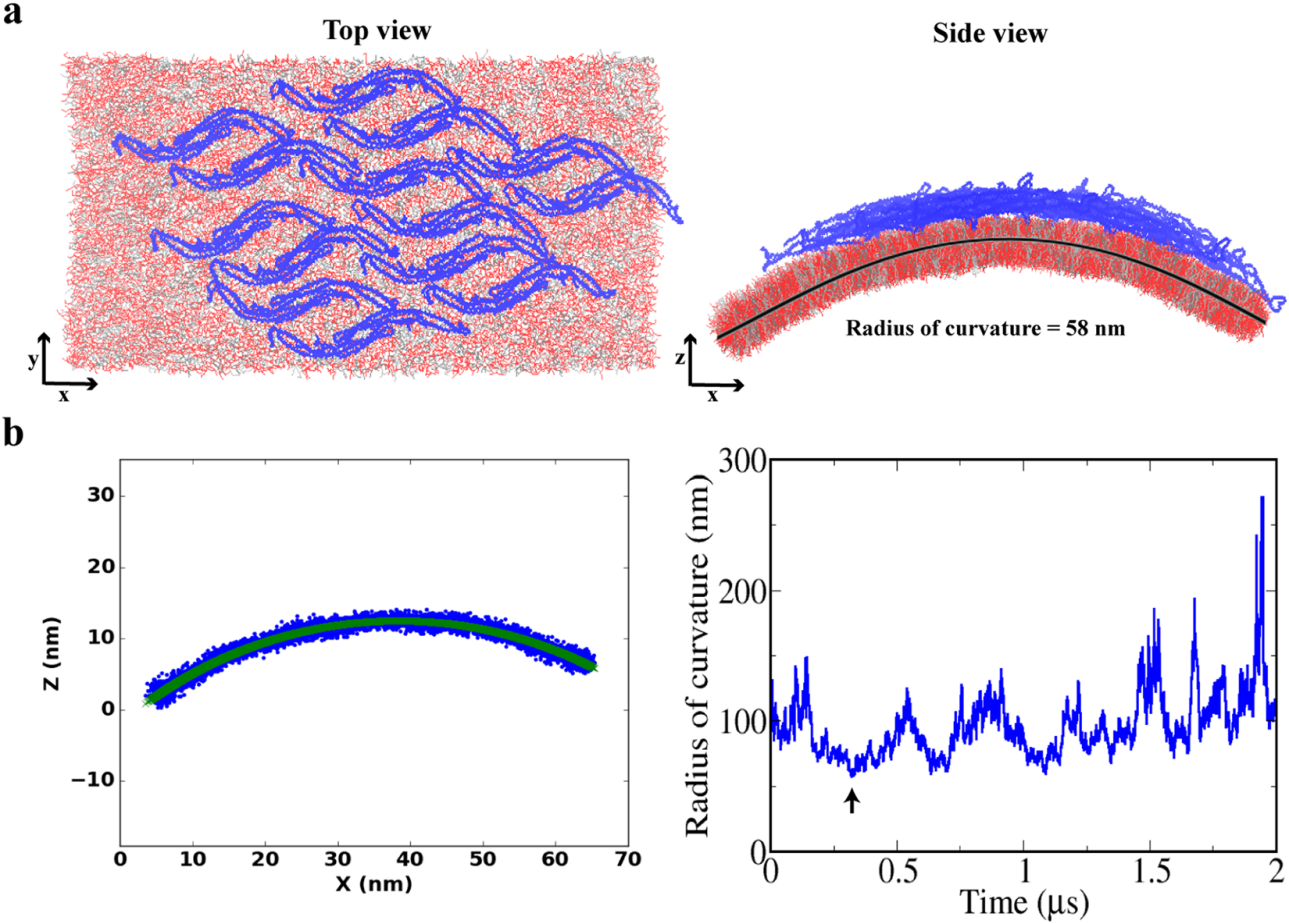
Membrane bending by regularly assembled Pacsin1. (**a**) Top and side views of the simulation snapshot of the membrane coated with the Pacsin1 assembly. (**b**) Fitting of *x-z* coordinates of innermost lipid beads (blue dots) by a circle (green line), which gives the radius of curvature (left panel). A time series of the obtained radius of curvature is plotted in the right panel. The arrow indicates the snapshot used in the left panel.

To show a direct link between Pacsin1 assembly and membrane remodelling, we modelled regularly assembled Pacsin1 on a tensionless membrane with free edges that run along the *y* direction. In the top view of Fig. 5a, the left and right vertical membrane edges are free, while the membrane is connected to its upper and lower periodic images. Because of the free edges, the membrane can bend freely in the *xz*-plane without a restoring force due to the periodic boundary condition (see Methods). The regularly assembled Pacsin1 was modelled based on the two-dimer complex structure with the lateral interaction described above. Initially, seven two-dimer complexes (14 dimers) were arranged in a similar way as that in the crystallographic lattice (P2_1_ lattice in PDB ID: 3HAI)^2^. The assembly indeed bent the membrane in a continuous manner during the 2 μs CG simulation (Fig. 5a). We estimated the curvature of the membrane by the least squares fitting of a circle to the *x-z* projection of the membrane after centering its peak position (Fig. 5b). The estimated radius of curvature fluctuated around 100 nm, down to ~60 nm. The estimated curvature is consistent with the wide featureless tubes from experiments^2^, where Pacsin1 induced tubulation with a diameter up to ~170 nm.

### Curvature sensing of Pacsin1 on a buckled membrane

**Figure 6:**
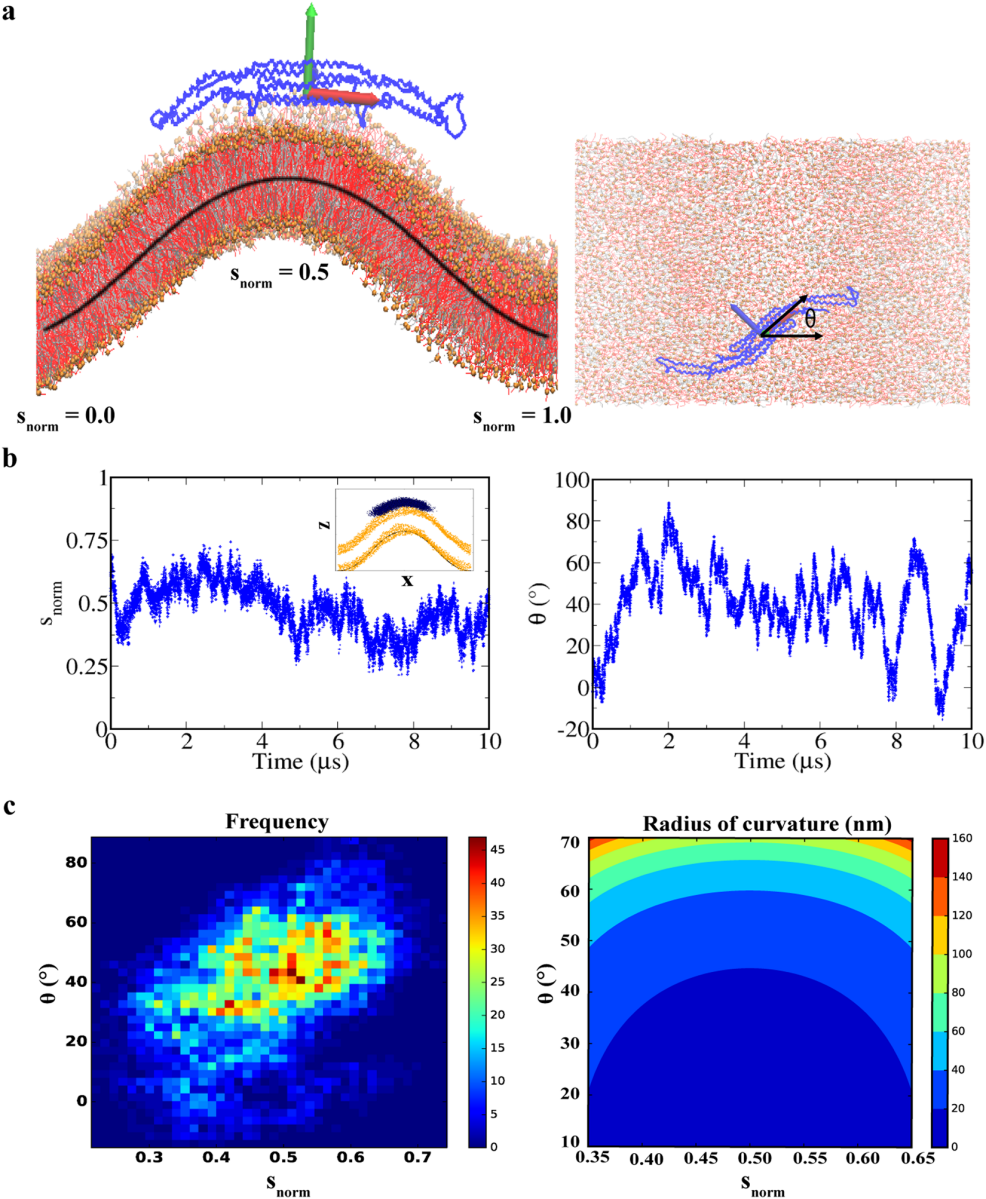
Pacsin1 on the buckled membrane. (**a**) Definitions of *s*_norm_ and *θ* are illustrated. (**b**) Time series of *s*_norm_ and *θ* during the 10 µs simulation are shown. The inset of the left panel shows the distribution of the centre of mass position of Pacsin1 in black points. (**c**) In the left panel, the frequency of the Pacsin1 position and orientation is plotted. In the right panel, analytical membrane curvature as a function of the position and orientation is plotted.

Curvature sensing of Pacsin1 is important for understanding how it assembles and induces membrane remodelling because Pacsin1 likely orients in a direction that has a specific membrane curvature. Simulations of Pacsin1 on a curved membrane provide a direct estimate of the curvature preference. Following the previous study on amphipathic peptides^41^, we simulated Pacsin1 on a buckled membrane^42,43^. On a buckled membrane, Pacsin1 can adapt itself to the membrane curvature specified by the binding position and angle (Fig. 6a). The centre of mass position of Pacsin1 on the arc length *s* (or the normalized arc length *s*_norm_) and the angle between the first principal axis of Pacsin1 and buckling direction *θ* were used for the binding position and angle, respectively. It has been shown that the membrane curvature can be analytically expressed as a function of *s* and *θ* (see Methods). With fixed *s*, the buckled membrane has the highest curvature at *θ* = 0 and zero curvature at *θ* = 90°.

From the 10 μs CG simulation of Pacsin1 on the buckled membrane, we calculated *s*_norm_ and *θ* (Fig. 6b). Then, we mapped them on the two-dimensional surface (Fig. 6c). The frequency plot shows that Pacsin1 was most populated around *s*_norm_ = 0.5 and *θ* = 45°. From the radius of curvature *R*(*s*, *θ*) plotted on the same two-dimensional surface, the most populated point corresponds to a radius of curvature of ~20 nm (diameter, ~40 nm). The free energy profile along the curvature shows a broad minimum with a radius of curvature of 17~33 nm (Fig. S5). Compared with the experimental tubule size generated by Pacsin1^2^, the size corresponding to the free energy minimum agrees well with the diameter of a striated tube (53 ± 18 nm). This result suggests that Pacsin1 orients perpendicular to the striated tube axis, which matches the preferred curvature of Pacsin1 and forms the striated pattern with a lateral interaction. Using the curvature-dependent binding free energy Δ*G*_bind_ = −2*k*_*B*_*T* at *R*_bind_ = 25 nm compared to a flat membrane (Fig. S5), the number density of Pacsin1 required to generate a tube with a diameter of 2*R*_bind_ was estimated to be ~0.008 nm^−2^ (see Methods). Note that the corresponding area per Pacsin1 ~125 nm^2^ is similar to an approximate area expected to be covered by Pacsin1 from its structure. Thus, it is expected that Pacsin1 dimers densely cover the tube surface. The vesicles with diameters of 35 ± 5 nm can also be explained by the preferred curvature of Pacsin1^2^.

## Discussion

The polymorphic membrane remodelling ability of Pacsin1 has been an unresolved mystery. Crystallographic studies have failed to correlate the structures with a variety of tubule diameters induced by Pacsin proteins^36^. Our AA simulations of the Pacsin1 dimer on the membrane, however, highlighted the flexibility of the banana-shaped structure. The crystal structure with a radius of curvature of ~25 nm became flatter with a radius of curvature of ~50 nm and larger during the simulations on the membrane (Fig. S1). This result suggests that Pacsin1 can adapt itself to a wide range of membrane curvatures and stabilize tubules with different diameters, merely from its internal structural flexibility. Thus, our result challenges the view that BAR domains are rigid objects, which is often assumed in theoretical or simulation studies. Flexibility can reduce the membrane-mediated interaction between two BAR proteins. Previously, it was reported that the meshless membrane simulation of flexible banana-shaped rods and analytical results for point-like rigid objects show excellent agreement in terms of the shape of the interaction potential but that the amplitude is smaller in the flexible rods^44^. Therefore, it is important to take flexibility into account for a quantitative understanding of membrane remodelling by the BAR domains.

The following CG MARTINI simulations revealed the curvature induction mechanism of Pacsin1. To induce membrane curvature, it is necessary for Pacsin1 proteins to assemble and bend the membrane in a collective way. Based on the two-dimer complex formed through the stable lateral interaction, we modelled regularly assembled Pacsin1 proteins. Then, we showed, in a direct way, that the assembly induces bending of the membrane. The estimated curvature is consistent with featureless wide tubes with diameters of 100~170 nm observed in experiments^2^. Our result is consistent with the membrane tubulation by the lattice assembly of other BAR domain proteins studied previously^27^. A helical Pacsin1 assembly can be formed by the lateral interaction. Such a helical assembly is crucial to inducing the formation of membrane tubes with a constant diameter^40^. The CG MARTINI simulations also revealed the curvature-sensing property of Pacsin1. From simulations of Pacsin1 on the buckled membrane, it was shown that Pacsin1 prefers a curvature that is consistent with the curvature of striated tubes and pearling vesicles observed in experiments^2^. Thus, our CG simulation results explain the experimentally observed featureless wide tubes in a direct way, while the striated tubes and vesicles are explained through the curvature preference of Pacsin1. The thin tubes with diameters of ~17 nm remain unresolved in this study.

The effect of the lipid composition on Pacsin1 binding is an important issue. The average lipid distributions near the membrane-bound Pacsin1 in the AA simulations show some localization of the different lipids, while they are completely uniform in the CG simulations (Fig. S6). It is likely that the relatively short timescale of the AA simulations results in the apparent localization. This point can be tested with longer AA simulations in the future.

## Methods

### All-atom simulations of the Pacsin1 membrane system

The initial structure of the Pacsin1 protein for MD simulations was taken from the X-ray crystal structure of the human Pacsin1 F-BAR domain (PDB ID: 3HAH)^2^. Missing residues of the Pacsin1 dimer structure (first monomer: T172-L191, second monomer: T172-K194) were modelled with MODELLER^45,46^. From the modelled structures, we selected a best model with the lowest value of the MODELLER assessment score, that is, the discrete optimized protein energy (DOPE)^47^. The initial coordinates of the mixed lipid bilayer (POPC 20%, POPE 20%, POPS 60%)^2^ were generated by the membrane builder tool of CHARMM-GUI^48,49^. This lipid composition was used in the experiment^2^. The Pacsin1 structure was aligned on the lipid bilayer using VMD^50^. Then, water molecules and ions were added to the system. The initial box sizes were 23 nm, 23 nm and 18 nm in the *x*, *y*, and *z* directions, respectively, and the total number of atoms was 909,109. The system was energy minimized with 5,000 steps of the steepest descent method. Then, 0.5 ns MD trajectories were conducted with positional restraints on heavy atoms of the protein under *NPT* conditions (T = 310 K and P = 1 bar) to equilibrate the systems. Then, all the restraints were eliminated, and production runs were conducted for 500 ns. Here, the Nose-Hoover thermostat, with a relaxation time of 1 ps, and the semi-isotropic Berendsen barostat, with a relaxation time of 2 ps, were used. The cutoff radii of the Lennard-Jones (LJ) and real-space electrostatic interactions were set to 1.2 nm. The MD time step was set to 2 fs. Hydrogen bond lengths were constrained using the LINCS algorithm. For long-range electrostatic interactions, the particle mesh Ewald (PME) method was used. The CHARMM36 force field was used for lipids and proteins^51^.

### MARTINI simulations of the Pacsin1 membrane system

MARTINI simulations of the Pacsin1-membrane system were performed using the MARTINI version 2.2 force field^18,52–54^ with additional elastic network potential for the protein^37^. We employed the GROMACS-2016 simulation package (www.gromacs.org) for the CG MD simulations^55^. The elastic network model was used to maintain protein secondary and tertiary structures with a spring constant of 500 kJ mol^−1^ nm^−2^ and lower and upper elastic bond cutoffs of 0.5 and 1.2 nm, respectively^37^. In our simulations, the upper elastic bond cutoff was explored (see main text). The Pacsin1 protein crystal structure (PDB ID 3HAH) was modelled using the parameters and protocols from the MARTINI community with the elastic network. First, the “martinize” script was used to generate the protein coarse-grained structure and topology^52^. Then, the proteins were merged with flat lipid bilayer, water molecules and ions using the script “insane”^56^. The lipid membrane consists of mixed lipids POPC, POPE and POPS (20%:20%:60%). An appropriate amount of solvent (CG water) was added along with 0.15 M Na^+^ and Cl^−^ ions to create a charge-neutral system similar to that in all-atom simulations. This brought the system size to 296,087 CG beads, and the initial box size was 60 nm, 30 nm and 20 nm in the *x*, *y* and *z* directions, respectively. We ran three independent simulations by changing only the protein elastic network (ELN) upper cutoff (ELN 0.9 nm, ELN 1.1 nm and ELN 1.2 nm). After energy minimization with 5,000 steps of the steepest descent method, production runs were performed at 300 K with separate temperature coupling for the solvent, lipids and proteins using the stochastic rescaling scheme (τ = 1 ps) and the Parrinello-Rahman semi-isotropic pressure coupling at 1 bar. A time step of *dt* = 20 fs was used. The reaction field electrostatics and Lennard-Jones potentials were shifted to zero at a cutoff distance of 1.1 nm.

For the membrane bending simulations, the membrane was prepared so that it had free edges at the ends in the *x* direction but closed edges with a periodic boundary at the ends in the *y* direction^27,57^. To achieve that, we constructed two flat bilayer membranes with periodic boundary box sizes of (60 × 30 × 20) nm and (70 × 30 × 25) nm, including proteins, water and ions. After energy minimization and short equilibration (1 ns), we replaced the membrane and proteins of the larger system with those of the smaller system. Then, we performed an energy minimization, equilibration (5 ns) and 2 *µ*s production run of the larger system with the replaced membrane and proteins. Anisotropic pressure coupling was applied in the *z* direction only.

### Buckled membrane simulations

The buckled membrane was prepared following a previous study^41^. We equilibrated a MARTINI flat membrane of 3742 lipids with 20% POPC, 20% POPE and 60% POPS, solvated with ~137,161 coarse-grained water molecules and neutralized with 0.15 M Na^+^ and Cl^−^ ion beads. This membrane was equilibrated for 25 ns in an *NPT* ensemble at 300 K and 1 bar, with pressure coupling applied semi-isotropically. After equilibration, the system was laterally compressed 10% in the *x* direction. This was done by scaling all *x* coordinates and the box size in the *x* direction to 90%. Thus, *L*_*x*_/*L* = 0.9, where original and rescaled box sizes in the *x* direction are *L* and *L*_*x*_, respectively. After the rescaling, the compressibility was set to zero in the *x* and *y* directions to keep the system size constant in those directions for subsequent simulation. Pressure coupling was then applied anisotropically in the *z* direction only. We then performed an energy minimization and a short equilibration run (50 ns) to let the bilayer buckle. Afterwards, we added one Pacsin1 dimer to the system. The elastic network with a force constant of 500 kJ mol^−1^ nm^−2^ and an upper cutoff of 1.2 nm was applied to the Pacsin1 protein. The protein was initially placed above the membrane surface but quickly attached to the membrane. We minimized the system and performed another 10 ns equilibration. Then, the production run was run for 10 *µ*s, where we collected data every 1 ns.

### Analysis of the Pacsin1 position and orientation on a buckled membrane surface

We used an analytic expression of the buckled membrane shape to locate the Pacsin1 position on the surface. It has been shown that the shape of a buckled membrane on the *x-z* plane (*x* is buckling direction) can be expressed using elliptic functions with a parametric variable of arc length *s* as^42,43^

1 $$\begin{array}{c}x(s)=2\lambda E[am[s/\lambda ,m],m]-s\\ z(s)=2\lambda \sqrt{m}(1-cn[s/\lambda ,m])\end{array}$$

where *E*[*a*, *m*], *am*[*a*, *m*] and *cn*[*a*, *m*] are the elliptic integral of the second kind, Jacobi amplitude, and Jacobi elliptic cosine function, respectively. The parameter *m*, the square of the elliptic modulus, was calculated from the series expansion of the compressed ratio *γ* as^43^

2 $$m=\gamma -\frac{1}{8}{\gamma }^{2}-\frac{1}{32}{\gamma }^{3}-\frac{11}{1024}{\gamma }^{4}\ldots $$

where *γ* = (*L* − *L*_*x*_)/*L* = 0.1 in this study. The total arc length *L* is related to the eliptic parameter *m* as $\frac{{L}_{x}}{L}=2\frac{E[m]}{K[m]}-1$, where *K*[*m*] and *E*[*m*] are complete elliptic integrals of the first kind and second kind, respectively. *λ* = *L*/4*K*[*m*] is called the characteristic length. Thus, once the compression ratio *γ* is given, the buckled shape is uniquely determined analytically (black curve in Fig. 6a). A SciPy library of special functions in Python was used to obtain the curve^58^.

To obtain the position of the Pacsin1 dimer on the buckled membrane, the membrane shape from the simulation snapshot was aligned to the analytical curve described above by centering the peak position and shifting the buckled membrane. Then, the centre of mass position of the Pacsin1 dimer on the normalized arc length coordinate *s*_norm_ ≡ *s*/*L* was calculated for every snapshot, where 0 ≤ *s*_norm_ ≤ 1. We also calculated the orientation of the Pacsin1 dimer *θ*, which is represented by the first principal axis of the molecule from the buckling direction (*x*) on the membrane (Fig. 6a).

### Curvature of the buckled membrane

Local curvature along the buckling direction *C*(*s*) as a function of arclength coordinate *s* is expressed as^43^

3 $$C(s)=\frac{d\psi }{ds}=\frac{\sqrt{2(\cos \,\psi -(1-2m))}}{\lambda }$$

where *ψ* is the tangent angle along the buckling direction and is expressed as $\psi (s)=2{\sin }^{-1}\{\sqrt{m}sn[s/\lambda ,m]\}$ with the Jacobi elliptic sine function *sn*[*s/λ*, *m*]. With angle *θ* from the buckling direction (*x*), the local curvature *C*(*s*, *θ*) is calculated from the principal curvatures *c*_1_ = *C*(*s*) and *c*_2_ = 0 as

4 $$C(s,\theta )={c}_{1}\,{\cos }^{2}\theta +{c}_{2}\,{\sin }^{2}\theta =C(s){\cos }^{2}\theta $$

The radius of curvature *R*(*s*, *θ*) = 1/*C*(*s*, *θ*) was calculated from *s*_norm_ and *θ* of Pacsin1 on the buckled membrane.

### Estimation of the Pacsin1 number density per area required to generate a tube

On the basis of the elastic theory of lipid bilayers^2,59^, the elastic energy cost per unit area required to generate a tube with a diameter of 2*R* from a flat membrane is

5 $${f}_{{\rm{tube}}}(R)=\frac{{k}_{c}}{2}\frac{1}{{R}^{2}}$$

where *k*_*c*_ is the membrane bending rigidity that is typically estimated as ~20 k_B_T^60^. With the binding energy of Pacsin1 at a radius of curvature *R*_bind_ compared to a flat membrane Δ*G*_bind_, the Pacsin1 number density per unit area required to generate a tube with a diameter of 2*R*_bind_ can be written as

6 $${\rho }_{{\rm{Pacsin1}}}={f}_{{\rm{tube}}}({R}_{{\rm{bind}}})/|\Delta {{G}}_{{\rm{bind}}}|$$

## Supplementary information

Supplementary Figures

## Data Availability

All data generated or analysed during this study are available from the corresponding author upon reasonable request.
