# Supplementary material for: Curvature induction and sensing of the F-BAR protein Pacsin1 on lipid membranes via molecular dynamics simulations
Source: Sci Rep. 2019 Oct 10;9:14557. doi: 10.1038/s41598-019-51202-z (PMC6787258; doi:10.1038/s41598-019-51202-z)
Supplement: Supplementary file 1 — Supplementary Figures [file 41598_2019_51202_MOESM1_ESM.docx]

**Supporting information for:**

**Curvature induction and sensing of the F-BAR protein Pacsin1 on lipid membranes via molecular dynamics simulations**

**Md. Iqbal Mahmood^1^, Hiroshi Noguchi^2^ and Kei-ichi Okazaki^1,*^**

^1^*Department of Theoretical and Computational Molecular Science, Institute for Molecular Science, National Institutes of Natural Sciences, Okazaki, 444-8585, Japan*

*^2^Institute for Solid State Physics, University of Tokyo, Kashiwa, Chiba 277-8581, Japan.*

* Correspondence should be addressed to: keokazaki@ims.ac.jp

This file contains Figures S1 – S6.


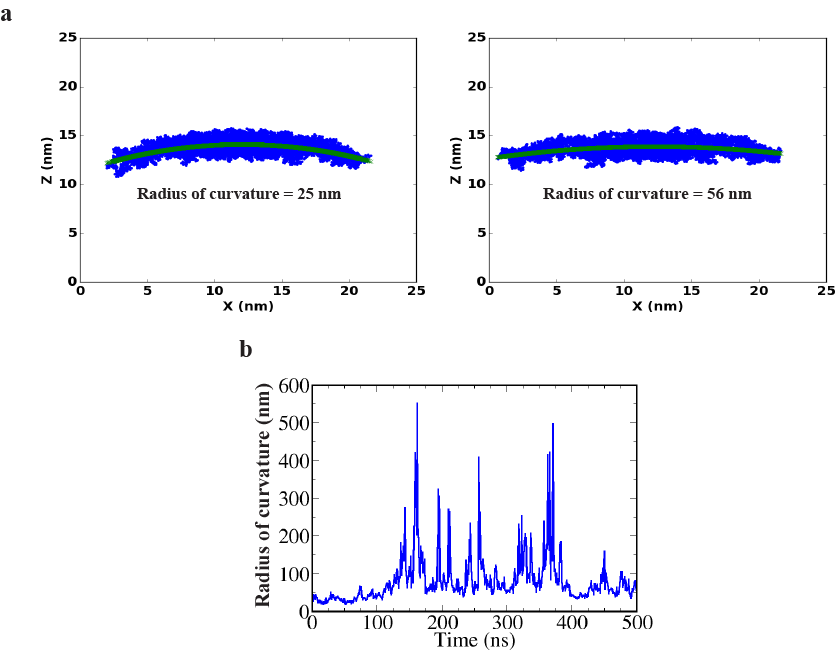


**Figure S1**: Pacsin1 dimer structure in the AA simulation. (a) Fitting of atom coordinates of Pacsin1 protein in side view (blue dots) by a circle (green line) provides a radius of curvature. The left and right panels show snapshots at 0 ns and 500 ns, respectively. (b) Time series of the obtained radius of curvature is plotted.


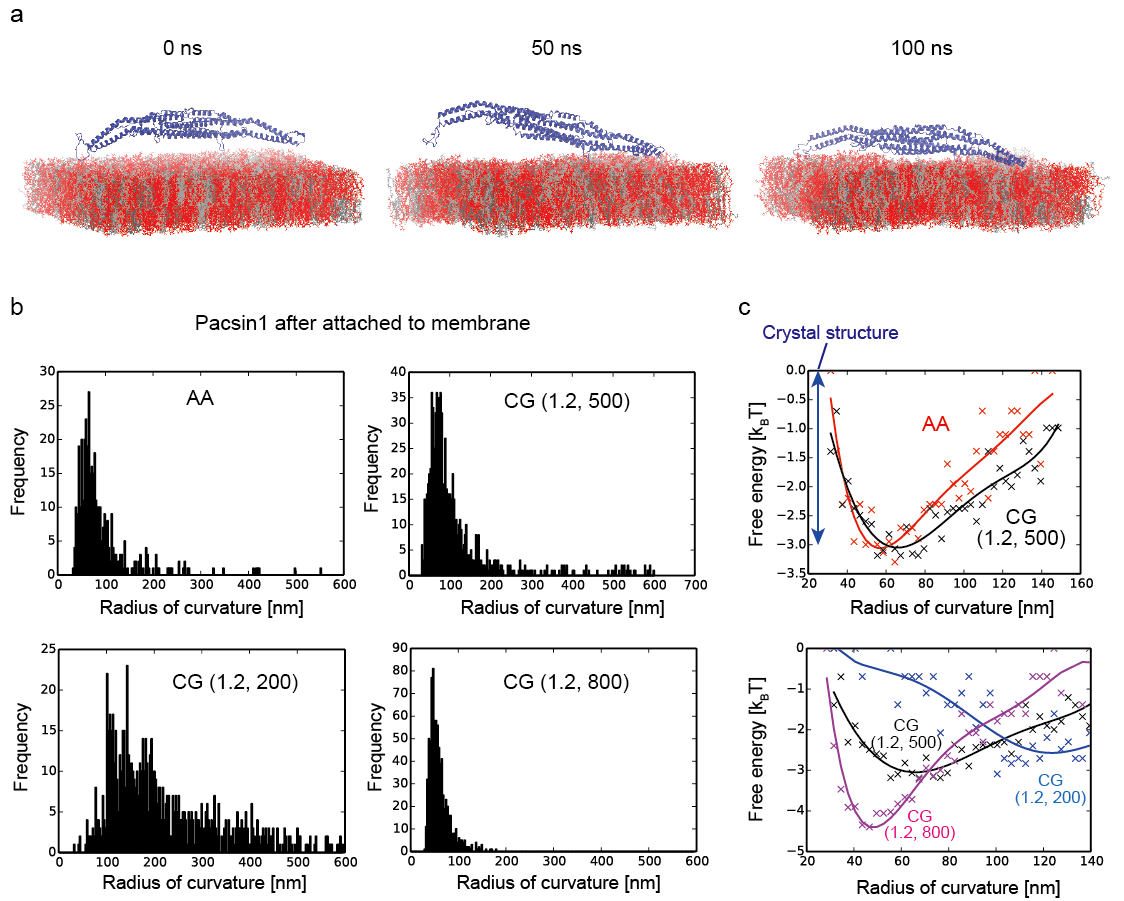


**Figure S2**: Pacsin1 structure after its attachment to the membrane in the AA and CG simulations. (a) Snapshots of Pacsin1 attaching on the membrane during the first 100 ns of the AA simulation are shown. (b) The frequency profiles of the radius of curvature of the Pacsin1 structure from the AA and CG simulations are shown. Numbers in parenthesis of the CG simulations represent the upper cutoff in nm and spring constant in kJ mol^-1^nm^-2^ of the elastic network potential. The first 20% of the trajectories were omitted to analyze the structure after its attachment to the membrane. (c) In the top panel, the free energy profiles along the radius of curvature from the AA simulation and CG simulation with the optimized elastic network are shown in red and black, respectively. The crosses represent free energies calculated from the frequency, $F\left( r \right)=-k_{\text{B}}T \ln p\left( r \right)$, where $p\left( r \right)$ is the frequency at a radius of curvature $r$. The data points were fitted with polynomial functions to get smooth curves shown in lines. The blue arrow represents the estimated free energy to bend the stable conformation back to the crystal structure. In the bottom panel, the free energy profiles from the CG simulations with the spring constants of 200, 500 and 800 kJ mol^-1^nm^-2^ are shown in blue, black and magenta, respectively.


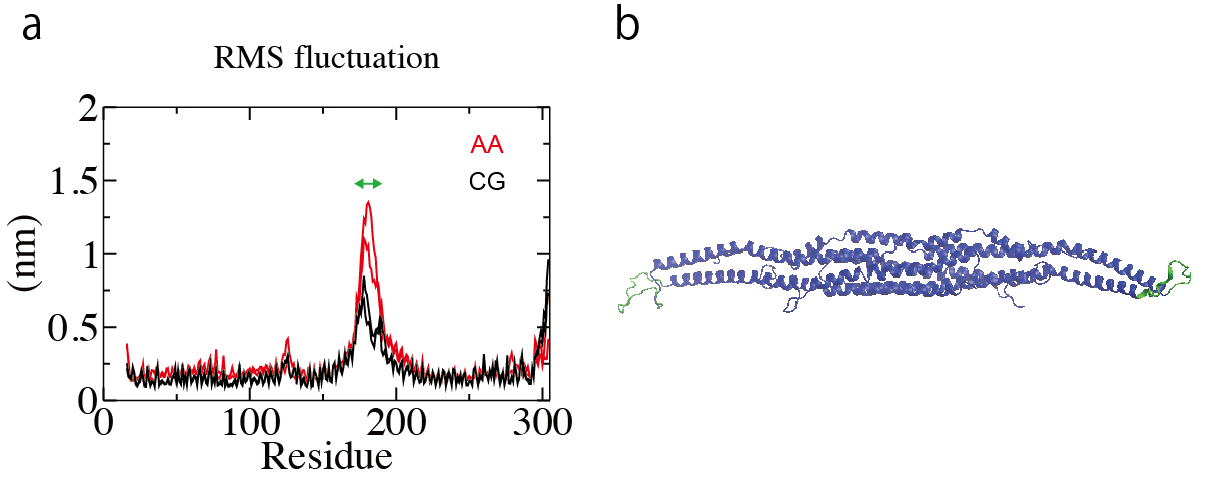


**Figure S3**: The root-mean-square fluctuations (RMSFs) of Pacsin1 from the AA and CG simulations. (a) The RMSFs of the two Pacsin1 monomers from the AA and CG simulations are plotted in red and black lines, respectively. The green arrow represents the tip-loop region. (b) The tip loops in the Pacsin1 structure are shown in green.


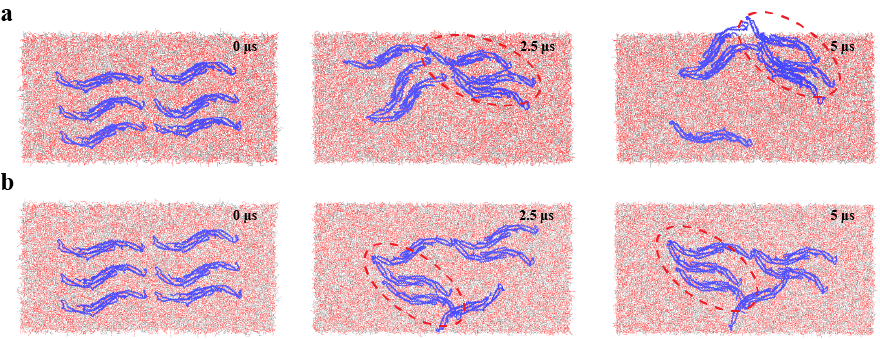


**Figure S4**: Assembly modes of Pacsin1 on a flat membrane. (a) and (b) Two independent 5 μs runs are shown. Spontaneous assembly of two Pacsin1 dimers through the lateral interaction is shown by dotted red circles.


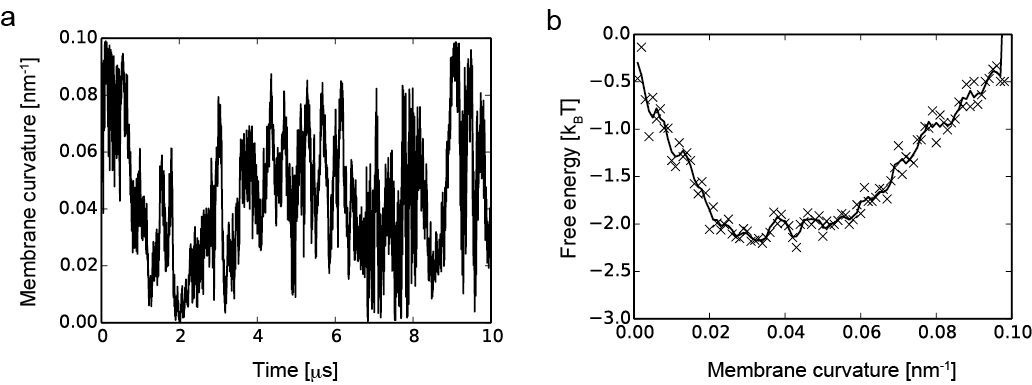


**Figure S5**: Membrane curvature sensed by Pacsin1 in the buckled membrane simulations. (a) Time series of the membrane curvature is plotted. (b) The Free energy profile along the membrane curvature is plotted. The crosses represent free energies calculated from the frequency, $F\left( c \right)=-k_{\text{B}}T \ln p\left( c \right)$, where $p\left( c \right)$ is the frequency at a membrane curvature $c$. The line represents a moving average of the data points.


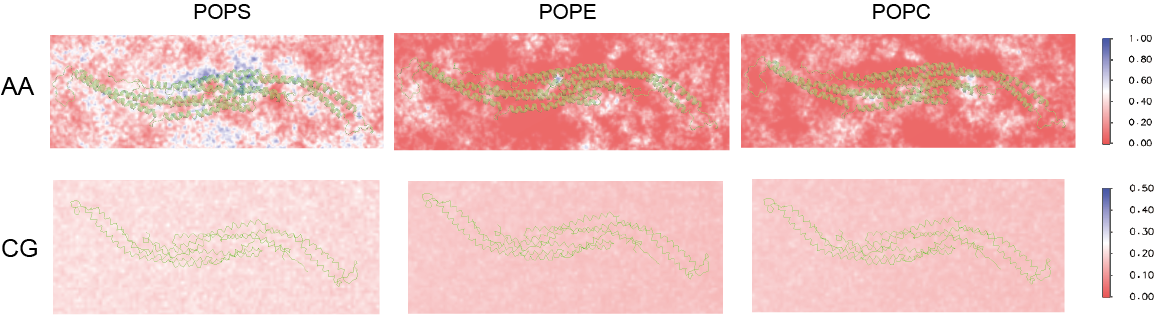


**Figure S6**: Distributions of the POPS, POPE and POPC lipids near the membrane-bound Pacsin1 are shown in left, center and right panels, respectively, from the AA (top) and CG (bottom) simulations. The average density in Å^-3^ was calculated for each lipid and shown in the volume-slice representation by VMD^1^. Red, white and blue colors represent small, intermediate and large densities, respectively. Pacsin1 is shown in green.

**References**

1. Humphrey, W., Dalke, A. & Schulten, K. VMD: Visual molecular dynamics. *J. Mol. Graph.* **14**, 33–38 (1996).
